# Cytotoxicity Evaluation of a New Set of 2-Aminobenzo[de]iso-quinoline-1,3-diones

**DOI:** 10.3390/ijms151222483

**Published:** 2014-12-04

**Authors:** Rashad Al-Salahi, Ibrahim Alswaidan, Mohamed Marzouk

**Affiliations:** 1 Department of Pharmaceutical Chemistry, College of Pharmacy, King Saud University, P.O. Box 2457, Riyadh 11451, Saudi Arabia; E-Mails: ralsalahi@ksu.edu.sa (R.A.-S.); ialsuwidan@ksu.edu.sa (I.A.); 2 Chemistry of Natural Products Group, Center of Excellence for Advanced Sciences, National Research Center, Dokki, Cairo 12622, Egypt

**Keywords:** cytotoxicity, HCT-116, Hep-G2, aminobenzo[de]isoquinoline-1,3-diones, doxorubicin

## Abstract

A new series of 2-amino-benzo[de]isoquinoline-1,3-diones was synthesized and fully characterized in our previous paper. Here, their cytotoxic effects have been evaluated *in vitro* in relation to colon HCT-116, hepatocellular Hep-G2 and breast MCF-7 cancer cell lines, using a crystal violet viability assay. The IC_50_-values of the target compounds are reported in µg/mL, using doxorubicin as a reference drug. The findings revealed that compounds **14**, **15**, **16**, **21** and **22** had significant cytotoxic effects against HCT-116, MCF-7 and Hep-G2 cell lines. Their IC_50_ values ranged between 1.3 and 8.3 μg/mL in relation to doxorubicin (IC_50_ ≈ 0.45–0.89 μg/mL). Therefore, these compounds could be used as templates for furthering the development and design of more potent antitumor agents through structural modification.

## 1. Introduction

Despite intensive efforts, and much progress being made to understand the biology of cancer, as well as the development of more effective anticancer chemotherapeutics, the disease still poses a serious threat to human health around the globe and the rates of response to existing antitumor drugs in clinical trials have remained largely unimproved. Consequently, the design of new lead structures to be employed as antitumor agents is always a fascinating challenge in the field of cancer chemotherapy and is a most urgently needed area of research in organic medicinal chemistry. Although a number of biologically active compounds have been investigated, many of them have been found to be unsuitable for therapeutic application due to their toxic, carcinogenic and mutagenic properties. Nowadays, it is possible to make structural modifications to newly synthesized active compounds in order to improve their therapeutic indices and reduce their toxicity.

Within the existing literature, several studies have reported that some naphthalimide derivatives play an essential role in the construction of many bioactive compounds, such as antitumor agents and histone deacetylase inhibitors (HDAC) [1,2,3,4,5,6,7,8,9,10,11,12]. In addition, a number of 1H-benzo[de]isoquinolin-1,3-diones have been shown to exhibit antimicrobial activity and have a high binding affinity towards the 5-HT_1A_ receptor that is expressed in CHO cells, as determined by fluorescence microscopy [11,12]. Furthermore, 1H-benzo[de]isoquinolin-1,3-dione-based anticancer drugs and potent HDAC inhibitors have formed an indispensable part in the development of antitumor agents [2,5]. For example, substituted naphthalimides containing the *N*-(2,2-dimethylaminoethyl) chain, best represented by mitonafide, amonafide and the dimer einafide, have been shown to possess significant anticancer properties [2,5,10]. Although promising findings have been obtained in this regard, many of these compounds were poorly studied, because of their sophisticated synthetic pathways [2]. Based on these points, the present work is an extension of our ongoing efforts towards designing and developing new active naphthalimide molecules. Therefore, in this study, we aimed to investigate a new series of 2-amino-benzo[de]isoquinolin-1,3-dione derivatives as antitumor agents.

## 2. Results

**Figure 1:**
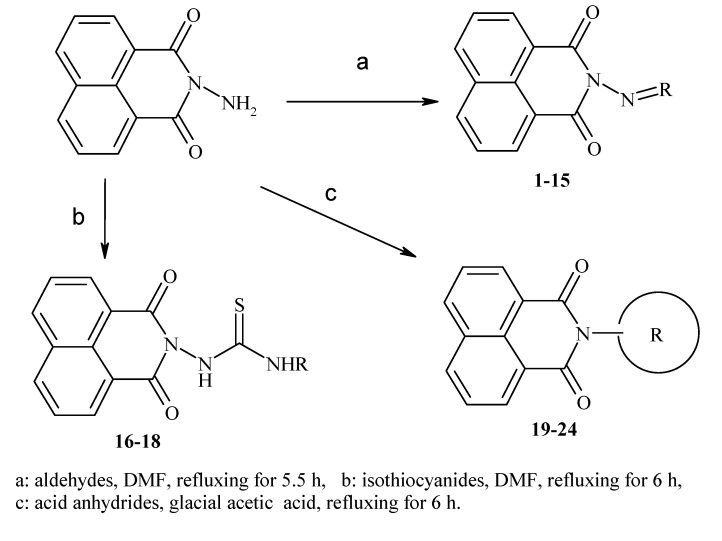
Main routes for synthesis of the target compounds **1**–**24**.

Previously, our work has reported on the synthetic methodology for the preparation of the target compounds **1**–**24**, as indicated in Table 1 and Figure 1 [13,14,15]. The *in vitro* cytotoxicity of target molecules **1**–**24** was evaluated against HCT-116, MCF-7 and Hep-G2 cells, using the crystal violet viability assay [16,17,18,19,20,21]. The IC_50_-values are summarized in Table 2 as common parameters for the cytotoxicity of the investigated compounds. Cytotoxicity of the target compounds to HCT-116, Hep-G2, and MCF-7 cells (IC_50_ values of 1.3–45.0 μg/mL) was comparable to that of doxorubicin (Table 2). Compounds **14**–**16**, **21** and **22** showed the highest cytotoxicity among all the compounds tested against HCT-116, MCF-7 and Hep-G2 cells (IC_50_ = 1.3–8.3 μg/mL) in comparison with their inactive parent compound (2-amino-benzo[de]isoquinolin-1,3-dione; IC_50_ > 50 μg/mL) and the reference drug doxorubicin (IC_50_ = 0.469, 0.892 and 0.426 μg/mL, respectively). However, compound **21** demonstrated less activity against Hep-G2 (IC_50_ = 19.8 μg/mL).

**Table 1:** Synthesized 2-amino-benzo[de]isoquinolin-1,3-dione derivatives (**1**–**24**).

Compound Number	R	Compound Number	R
**1**	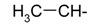	**13**	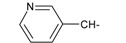
**2**	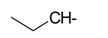	**14**	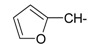
**3**	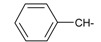	**15**	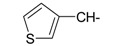
**4**	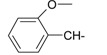	**16**	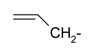
**5**	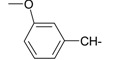	**17**	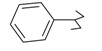
**6**	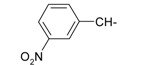	**18**	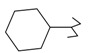
**7**	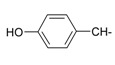	**19**	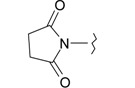
**8**	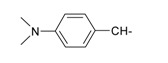	**20**	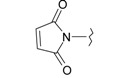
**9**	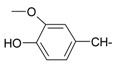	**21**	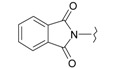
**10**	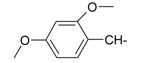	**22**	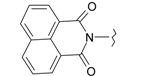
**11**	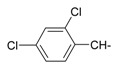	**23**	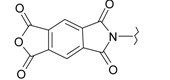
**12**	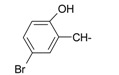	**24**	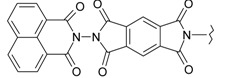

## 3. Discussion

Using the crystal violet assay [16,17,18,19,20,21], we found that compounds **4**, **7**, **10** and **17** exhibited good cytotoxicity against the HCT-116 cell line, with IC_50_ values of 16.3, 16.9, 18.2 and 16.7 μg/mL respectively, in relation to the most active compounds (**14**–**16**, **21** and **22**). Compounds **3**, **4**, **10** and **20** (IC_50_ = 14.8, 19.7, 12.2 and 19.0 μg/mL, respectively) showed good cytotoxicity against Hep-G2, as did compounds **4**, **9**, **10**, **17** and **13** (IC_50_ = 17.9, 11.2, 19.1, 16.3 and 19.7 μg/mL) against the MCF-7 cell line.

Compounds **1**, **2**, **5**, **11**, **13** and **24** were found to be less active against all investigated cell lines, except for compound **13** (IC_50_ = 19.7 μg/mL), which demonstrated moderate activity against MCF-7. Our results show that compounds **14**–**16**, **21** and **22** had the lowest IC_50_ values against the three cancer cells employed in relation to doxorubicin.

Based on the results in Table 2, it can be concluded that the type of substituent attached to the 2-amino group is a major determinant of the pharmacological properties of the parent structure (2-amino-benzo[de]isoquinolin-1,3-dione). Accordingly, in the case of compounds **14** and **22**, the cytotoxicity increased in the order of MCF-7 > Hep-G2 > HCT-116. Similarly, **15** and **16** demonstrated the order of Hep-G2 > HCT-116 > MCF-7 and MCF-7 > HCT-116 > Hep-G2 respectively in the cytotoxicity profile. This could be attributed to the magnitude and conformation of the hetero aldehyde, isothiocyanide and acid anhydride substituents, which may play a substantial role in the cytotoxic effects and the selectivity of these compounds. Throughout this study, we noticed that the chemical structures of the aldehyde substituents were varied in terms of cytotoxicity. Therefore, elongation of the aliphatic C-chain in aldehyde **2** could be considered to be the factor responsible for the slight increase in activity against Hep-G2 and MCF-7 cells. In the case of the aromatic aldehydes, variations in the positions of substitution on the benzyl ring affected their cytotoxicity, and compounds **4**, **7** and **10** showed the highest activity among all the aromatic aldehyde derivatives examined. Additionally, compound **11** displayed the lowest activity against all cell lines, and compound **13** had lower activity against HCT-116 and Hep-G2 compared to the former group of aldehyde derivatives. Concerning isothiocyanide derivatives, **16** was more active than **17**; however, compound **18** exerted the lowest activity against all examined cell lines. Moreover, acid anhydride derivatives demonstrated cytotoxic effects in the order of **22** > **21** > **23** > **20** > **19** > **24**, which could be due to the structural variation of the acid anhydride moiety. These results provide evidence that the characteristic chemical features of aldehyde, isothiocyanide and acid anhydrides are key factors for their cytotoxicity and could, therefore, play a useful role in elucidating the mechanisms of action in relation to the target products in future research programs. Regarding sensitivity of cell lines, HCT-116 appeared to be the most sensitive cell line to the antiproliferative activity of the tested compounds, since it responded to eight compounds, followed by MCF-7 and Hep-G2 which responded to seven and six compounds, respectively. It is noteworthy to point out that the higher sensitivity of all cell lines noticed towards compounds **14**, **15**, **16** and **22** followed by **7**, **17** and **21**.

**Table 2:** Cytotoxicity of the target compounds **1**–**24** (IC_50_, μg/mL).

Compound Number	IC_50_ (µg/mL)		
	HCT-116	Hep-G2	MCF-7
1	37.3	37.0	42.1
2	41.9	35.4	33.8
3	27.9	14.8 **	30.4
4	16.3 **	19.7 *	17.9 *
5	36.1	30.9	30.1
6	31.2	24.4 *	27.9
7	16.9 **	12.2 **	25.8
8	45.0	47.1	43.5
9	20.3 *	21.2 *	11.2 **
10	18.2 *	17.0 *	19.1 *
11	40.2	47.6	35.0
12	41.1	44.1	38.2
13	40.0	>50	19.7 *
14	3.5 ***	2.5 ***	1.3 ***
15	3.1 ***	2.7 ***	3.7 ***
16	6.7 ***	8.3 ***	6.1 ***
17	16.7 **	20.6	16.3 **
18	41.2	43.6	40.4
19	35.9	39.4	23.7 *
20	24.7 *	19.0 *	23.7 *
21	11.0 **	19.8 *	5.0 ***
22	5.3 ***	4.4 ***	3.5 ***
23	23.3 *	24.7	21.4 *
24	>50	27.9 *	>50
Parent	>50	>50	>50
Doxorubicin	0.469	0.892	0.426

*** *p* < 0.005 highly significant; ** *p* < 0.01 significant; * *p* < 0.5 non significant relative to the reference drug doxorubicin.

## 4. Experimental Section

### 4.1. Cell Lines

Human colon (HCT-116), breast (MCF-7) and hepatocellular (Hep-G2) carcinoma cells were obtained from the American Type Culture Collection (ATCC, Rockville, MD, USA). The cells were grown in RPMI-1640 medium, supplemented with 10% inactivated foetal calf serum and 50 µg/mL gentamycin. The cells were maintained at 37 °C in a humidified atmosphere with 5% CO_2_ and were sub-cultured two to three times per week.

### 4.2. Evaluation of Antitumor Activity

Cytotoxicity of all compounds (**1**–**24**; Table 1) was tested in MCF-7, HCT-116 and Hep-G2 cells. All the experiments and data concerning the cytotoxicity evaluation were performed and analyzed by Mahmoud M. Elaasser (Regional Center for Mycology and Biotechnology RCMB, Al-Azhar University, Cairo, Egypt). The cells were grown in growth medium (RPMI-1640) as monolayers and supplemented with 10% inactivated foetal calf serum and 50 µg/mL gentamycin. The monolayers were then washed with sterile, phosphate-buffered saline (0.01 M, pH 7.2) followed by treatment with 100 μL aliquots of the test samples diluted in fresh maintenance medium and incubated at 37 °C. Untreated cells served as controls. Three independent experiments were performed each containing six replicates for each concentration from eight 2-fold serial concentrations of the tested samples (0.39, 0.78, 1.56, …, 50 μg/mL). The cytotoxic effects of the tested compounds were then measured using crystal violet staining viability assay [22,23]. Briefly, after 24 h incubation at 37 °C and 5% CO_2_, the medium was removed and replaced with 100 μL 0.5% crystal violet solution. After 20-min incubation at room temperature, the plates were washed with phosphate buffered saline and viable crystal violet-stained cells were lysed using 33% glacial acetic acid solution. Absorbance of the glacial acetic acid solution in each well was then measured at 590 nm using a microplate reader (SunRise, TECAN, Inc, Morrisville, NC, USA). Using this colorimetric procedure, tested compound-mediated cell lysis and the cytotoxic effect of doxorubicin (used as a positive control) were measured and compared to the viability of untreated cells according to the following calculation:

the percentage of cell viability = [1 − (ODt/ODc)] × 100%
(1)

where ODt is the mean optical density of wells treated with the tested sample and ODc is the mean optical density of untreated cells. The tested compounds were compared using the IC_50_ value, *i.e.*, the concentration of an individual compound leading to 50% cell death that was estimated from graphical plots of surviving cells vs compound concentrations.

### 4.3. Statistical Analysis

The data were analysed statistically by using one-way analysis of variance (ANOVA) [24]. The difference was considered significant where *p* < 0.005 or *p* < 0.01 and was considered non-significant when *p* > 0.5.

## 5. Conclusions

In conclusion, the data obtained clearly indicates that some of the compounds tested exhibited good cytotoxic activity. This study has revealed that compounds **14**, **15**, **16** and **22** are the most active agents. Detailed biological studies on the molecular mechanisms of action of these derivatives are in progress.
